# Author’s Reply: Mental Health Problems Among Children and Adolescents From a Sports Sociology Perspective

**DOI:** 10.2196/62775

**Published:** 2024-07-03

**Authors:** Grace Aldridge

**Affiliations:** 1 Turner Institute for Brain and Mental Health School of Psychological Sciences Monash University Clayton Australia

**Keywords:** parenting interventions, technology, sports sociology, child mental health, adolescent mental health, adverse childhood experiences, systematic review, intervention, digital technology, parenting, parenting program, engagement, support

We thank the authors for their thoughtful response and comments on our systematic review [1]. As discussed in our paper, comprehensive evaluations (including quantitative syntheses) of engagement strategies and outcomes in technology-assisted parenting programs underline a significant gap in the parenting and child mental health literature. This gap is especially notable in the context of the COVID-19 pandemic, highlighting the clear utility technology has for maintaining or, at times, improving parents’ engagement with services and programs to support their young person’s mental health. While heterogeneous definitions, measurement of engagement, and a lack of engagement outcome data were indeed limitations to our review findings, we hope that our work provides a taxonomy of engagement strategies and measures that can help future work in the field to overcome these limitations.

The authors proposed that the field of sports sociology can play an important role in the prevention of mental health problems among adolescents. In particular, they outlined how family physical activity can promote a positive family environment, and how parenting behavior that supports and encourages family physical activity can promote young people’s mental and physical health. Given the strong link between adverse childhood experiences (ACEs) and poorer mental and physical health outcomes, we agree that exploring theories and methods from a sports sociology perspective is a worthwhile avenue for innovative solutions to preventing and reducing the impact of ACEs on young people’s mental health. Some technology-assisted parenting programs included in our study’s analysis included a physical activity component, and we wish to offer the authors some insights that may inform future research.

Our review included findings from the “Grow” parenting program [2] that aims to teach parents about key factors for healthy child development (such as physical activity) and has demonstrated effectiveness in helping parents increase child outdoor playtime and meet health recommendations. Parents appeared to engage well with both face-to-face and online versions of the “Grow” program; however, greater parental satisfaction with the face-to-face version led the authors to propose hybrid program models for future research to consider [3]. Our review also included findings from the web-based “Parenting Resilient Kids” program [4] that includes a module on encouraging healthy habits such as promoting regular physical exercise. While parenting behaviors specific to physical activity were not independently evaluated, this program demonstrated effectiveness in improving target parenting behaviors covered across all modules selected by parents. Furthermore, parents’ engagement with the program was shown to predict child health-related quality of life outcomes at the 12-month follow-up [5].

We believe these findings highlight technology-assisted parenting programs as an innovative solution for integrating sports sociology in efforts to prevent young people’s mental health problems. Given the link between parents’ engagement with these programs and program outcomes, designing engaging programs is important for these solutions to be effective. We therefore propose that the authors and those contributing to future research in this space consider drawing on the taxonomy of engagement strategies and measures presented in our review when designing such programs, to conceptualize engagement that is evidence-informed and facilitates evaluation.

## Abbreviations

ACE: adverse childhood experience
